# Caregiver exposure to malaria social and behaviour change messages can improve bed net use among children in an endemic country: secondary analysis of the 2015 Nigeria Malaria Indicator Survey

**DOI:** 10.1186/s12936-019-2750-x

**Published:** 2019-04-06

**Authors:** Kirsten Zalisk, Samantha Herrera, Uwem Inyang, Audu Bala Mohammed, Perpetua Uhomoibhi, Yazoumé Yé

**Affiliations:** 1 MEASURE Evaluation, ICF, 530 Gaither Road, Suite 500, Rockville, MD 20850 USA; 2 Save the Children, 899 North Capitol Street NE #900, Washington, DC 20002 USA; 3 President’s Malaria Initiative/United States Agency for International Development, Plot 1075 Diplomatic Drive, Central District Area, Abuja, Nigeria; 4 National Malaria Elimination Programme, Federal Ministry of Health Nigeria, 1st Floor, Abia Plaza, 1 Avenue, Cadastral Zone A0, Central Business District, Abuja, Nigeria

**Keywords:** Malaria prevention, Social and behavior change interventions, Insecticide-treated net use, Nigeria, Sub-Saharan Africa

## Abstract

**Background:**

To reduce the malaria burden in Nigeria, the National Malaria Strategic Plan (NMSP) 2014‒2020 calls for the scale-up of prevention and treatment interventions, including social and behaviour change (SBC). SBC interventions can increase awareness and improve the demand for and uptake of malaria interventions. However, there is limited evidence supporting the implementation of SBC interventions to improve key malaria behaviours, such as insecticide-treated bed net (ITN) use, among children in Nigeria.

**Methods:**

Using data from 2015 Nigeria Malaria Indicator Survey, this study used multiple logistic regression to assess the relationship between caregiver exposure to malaria messages and ITN use among children under five.

**Results:**

Caregiver exposure to ITN-related messages was significantly associated with ITN use among children under five (odds ratio [OR] = 1.63, p < 0.001).

**Conclusions:**

The results suggest that caregiver exposure to topic-specific SBC messages improves the use of ITNs among children. Given these results, Nigeria should strive to scale up SBC interventions to help increase ITN use among children in line with the objectives of the NMSP. Further evidence is needed to determine which SBC interventions are the most effective and scalable in Nigeria.

## Background

Malaria is a major health issue in Nigeria. In 2016, there were an estimated 57.3 million malaria cases and 100,700 malaria deaths [1]. In 2015, approximately 21% of deaths, 60% of outpatient visits, and 30% of hospitalizations of children under five were caused by malaria [2]. To address the malaria burden, Nigeria is expanding key malaria interventions. The country’s National Malaria Strategic Plan (NMSP) 2014‒2020 aims to transition the focus from malaria control to malaria elimination, with the goal of achieving pre-elimination status and reducing malaria-related deaths to zero by 2020 [3]. To reach this goal, the strategic plan calls for the scale-up of prevention strategies, including universal long-lasting insecticide-treated net (LLIN) coverage.

Although coverage of key malaria interventions is improving, large gaps remain, especially among children under five, and further effort is needed to reach the country’s 2020 targets [4]. According to the 2015 Nigeria Malaria Indicator Survey (NMIS), approximately two-thirds of households in Nigeria own at least one insecticide-treated net (ITN), but only 44% of children under five used an ITN the night before the survey (2015 NMSP target: 50%) [4]. Notably, more than 90% of ITNs are obtained free-of-charge through campaigns or through regular distribution during antenatal care or child immunization visits or at government health facilities in Nigeria [4].

Social and behaviour change (SBC) interventions are also an important component of Nigeria’s NMSP and are seen as integral to achieving its intervention coverage targets by creating awareness and improving demand for and the uptake of prevention and treatment interventions. Malaria SBC interventions are widely employed in the country. For example, mass ITN distributions are accompanied by SBC messaging [5, 6]. Programme implementers have used SBC interventions that involve national mass media messaging, interpersonal communication at the community level, and multi-channel approaches in their programme areas [7–9].

Despite widespread implementation of SBC interventions in malaria programming, there is limited evidence of their effectiveness for improving ITN use specifically among children under five and specifically in Nigeria [5, 6, 10–19]. Nevertheless, several studies call for the implementation of SBC interventions to improve the uptake of malaria interventions or behaviours among children under five [20–29]. Given this context and Nigeria’s emphasis on SBC interventions in its NMSP, this study assessed the relationship between caregiver exposure to malaria SBC messages and the use of ITNs among children under five.

## Methods

### Study design

This cross-sectional study was a secondary analysis of 2015 NMIS data to assess the relationship between mother or caregiver (hereafter referred to as “caregiver”) exposure to malaria SBC messages and ITN use among children under five.

### Data

The 2015 NMIS used a cluster-based sampling design to select a nationally representative sample of more than 8000 households from 329 clusters throughout the country. A total of 7745 selected households agreed to participate in the survey (response rate: 98.8%). In each household, all women between the ages of 15 and 49 were eligible to be interviewed, and 8034 women agreed to participate (response rate: 99.1%). Survey fieldwork took place in October and November 2015, immediately following the end of the rainy season, during the period when malaria transmission peaks. Further details on the survey methodology are provided in the survey final report [4].

### Data analysis

The authors used multiple logistic regression [30, 31] to assess the relationship between caregiver exposure to at least one ITN-specific malaria message and ITN use among children under five. The outcome variable was children under five who slept under an ITN the night preceding the survey. The exposure variable was caregivers of children under five who were exposed to at least one ITN-related message in the 6 months preceding the survey. Exposure to a message related to ITN use was defined as recall of at least one of the following two messages in the past 6 months: “*sleeping inside a mosquito net is important*” or “*who should sleep inside a mosquito net*”.

The model adjusted for select variables that could potentially confound the relationship between the outcome and exposure variables. These covariates were age and sex of the child, caregiver’s educational attainment, place of residence (i.e., urban or rural), region of the country, household wealth quintile [32], household ownership of a radio, household ownership of a television, and adequate number of ITNs in household—defined as the household owning at least one ITN per every two household members. The model was restricted to include one randomly selected eligible child per household to avoid cluster effects. The model was also restricted to include only children who slept in the household the night preceding the survey in households that owned at least one ITN. All analyses were done using Stata version 14 (StataCorp LLC, College Station, Texas, USA).

## Results

### ITN use among children under five by background characteristics

Insecticide-treated net use among children under five did not vary by child sex, child age, or household ownership of a radio, but it did vary by a number of characteristics. ITN use increased as the wealth quintile decreased, with 40.5% of children under five having used an ITN in the highest wealth quintile, and 69.9% of children under five having used an ITN in the lowest wealth quintile (p < 0.0001). ITN use was higher among children who lived in households with an adequate number of ITNs (69.1%, p < 0.0001), among children whose caregivers were exposed to an ITN-related message (55.9%, p = 0.0004), among children from rural areas (62.0%, p = 0.0005), among children from the North Central (60.7%), North East (61.0%) and North West (70.5%) regions (p < 0.0001), and among children living in households that did not own a television (66.3%, p < 0.0001) (Table 1).

**Table 1:** ITN use among children under five by background characteristics

Background characteristic	N	% (95% CI)	Chi square p-value
Caregiver exposed to at least one ITN message^a^			*0.0004*
No	1773	55.9 (52.7–59.1)	
Yes	302	67.9 (61.4–73.8)	
Sex of child			0.8894
Male	1057	58.1 (54.4–61.7)	
Female	1018	57.7 (53.5–61.9)	
Age of child			0.2991
0–11 months	285	54.5 (46.4–62.3)	
12–23 months	603	57.9 (52.9–62.8)	
24–35 months	532	62.2 (57.1–67.0)	
36–47 months	384	54.1 (51.6–62.9)	
48–59 months	271	57.9 (46.6–61.4)	
Caregiver’s educational attainment			*< 0.0001*
No formal education	890	67.7 (63.3–71.9)	
Primary education	365	54.9 (48.7–60.9)	
Secondary or higher education	820	47.9 (43.6–51.9)	
Place of residence			*0.0005*
Urban	728	49.9 (44.8–55.1)	
Rural	1347	62.0 (58.0–65.9)	
Wealth quintile			*< 0.0001*
Highest	393	40.5 (35.2–46.1)	
Fourth	411	47.5 (41.6–53.6)	
Middle	428	60.0 (54.5–65.3)	
Second	444	67.3 (60.8–73.2)	
Lowest	399	69.9 (64.0–75.3)	
Region			*< 0.0001*
North Central	363	60.7 (53.5–67.5)	
North East	424	61.0 (55.1–66.6)	
North West	596	70.5 (64.6–75.8)	
South East	184	33.3 (24.0–44.1)	
South South	250	45.4 (36.9–54.3)	
South West	258	41.7 (35.8–47.8)	
Adequate number of ITNs in household^b^			*< 0.0001*
No	1352	51.6 (48.3–55.0)	
Yes	723	69.1 (64.4–73.5)	
Household owns radio			0.2346
No	814	59.7 (55.2–64.1)	
Yes	1261	56.7 (53.2–60.2)	
Household owns television			*< 0.0001*
No	1101	66.3 (61.9–70.3)	
Yes	974	47.4 (43.6–51.2)	

In Nigeria almost all ITNs are long-lasting insecticide-treated net (LLINs). The authors used the term ITN because the analysis was conducted using data on ITNs, which include LLINs and ITNs that have been soaked with insecticide within the past 12 months

*ITN* insecticide-treated bed net, *CI* confidence interval

p-values that are significant at the 0.05 level are italic

^a^During the 6 months preceding the survey

^b^Household owns at least one ITN for every two household members

### Caregiver exposure to ITN messages and ITN use among children under five

The odds of ITN use were 1.6 times higher among children whose caregivers were exposed to at least one ITN message in the 6 months preceding the survey compared with children whose caregivers were not exposed to at least one ITN message in the 6 months preceding the survey (odds ratio [OR] = 1.63, p < 0.001). ITN use was significantly associated with residence, wealth quintile, region, adequate number of ITNs in household, and household ownership of a radio. Significant associations were not found between ITN use and sex or age of the child, caregiver’s educational attainment, or household ownership of a television (Table 2).

**Table 2:** Caregivers’ exposure to ITN messages and ITN use among children under five

Background characteristic	Odds ratio (95% CI)	p-value
Predictor		
Caregiver exposed to at least one ITN message^a^		
No	1.00 (reference)	–
Yes	1.63 (1.24–2.15)	*< 0.001*
Covariates		
Sex of child		
Male	1.00 (reference)	–
Female	0.95 (0.79–1.15)	0.619
Age of child		
0–11 months	1.00 (reference)	–
12–23 months	0.96 (0.71–1.30)	0.809
24–35 months	1.05 (0.77–1.43)	0.760
36–47 months	0.86 (0.62–1.19)	0.359
48–59 months	0.87 (0.61–1.25)	0.465
Caregiver’s educational attainment		
No formal education	1.00 (reference)	–
Primary education	0.92 (0.69–1.22)	0.556
Secondary or higher education	1.03 (0.77–1.37)	0.844
Place of residence		
Urban	1.00 (reference)	–
Rural	0.71 (0.55–0.93)	*0.011*
Wealth quintile		
Highest	1.00 (reference)	–
Fourth	1.48 (1.08–2.04)	*0.015*
Middle	2.45 (1.64–3.65)	*< 0.001*
Second	3.40 (2.08–5.55)	*< 0.001*
Lowest	3.40 (2.00–5.77)	*< 0.001*
Region		
North Central	1.00 (reference)	–
North East	0.80 (0.59–1.09)	0.153
North West	1.26 (0.93–1.71)	0.138
South East	0.34 (0.23–0.50)	*< 0.001*
South South	0.68 (0.48–0.96)	*0.029*
South West	0.56 (0.39–0.80)	*0.001*
Adequate number of ITNs in household^b^		
No	1.00 (reference)	–
Yes	2.23 (1.82–2.72)	< 0.001
Household owns radio		
No	1.00 (reference)	–
Yes	1.25 (1.02–1.54)	0.032
Household owns television		
No	1.00 (reference)	–
Yes	1.08 (0.80–1.45)	0.634

In Nigeria almost all ITNs are long-lasting insecticide-treated net (LLINs). The authors used the term ITN because the analysis was conducted using data on ITNs, which include LLINs and ITNs that have been soaked with insecticide within the past 12 months

*ITN* insecticide-treated bed net, *CI* confidence interval

p-values that are significant at the 0.05 level are italic

^a^During the 6 months preceding the survey

^b^Household owns at least one ITN for every two household members

## Discussion

This study assessed the relationship between caregiver exposure to malaria SBC messages and ITN use among children under five. The results suggest that caregiver exposure to ITN-related messages in the preceding 6 months has a positive effect on ITN use among children under five. Approximately two-thirds of households in Nigeria own at least one ITN, of which the majority are obtained through distribution campaigns [4] that usually include an SBC intervention to encourage regular, consistent, and proper use of the nets [5, 6]. ITNs are also obtained during antenatal care or child immunization visits, which offer opportunities for healthcare providers to share SBC messages specifically with caregivers of young children to ensure that they understand the benefits of their children sleeping under an ITN. The two most common sources of malaria messages are the radio and television [4], which can supplement messages provided during distribution campaigns and healthcare visits.

Other studies assessing the relationship between exposure to a malaria SBC intervention or message and ITN use among children have shown mixed results [11, 13, 16, 19]. For example, studies in Cameroon and Ghana found significant positive associations between caregiver exposure to malaria messages and ITN use in children [13, 19], but two studies in Zambia did not find a significant association between caregiver exposure to SBC interventions and ITN use among children [11, 16]. Although a limited number of studies explore ITN use among children in relation to caregiver exposure to relevant SBC messages, more studies look at ITN use among other household members in relation to exposure to SBC messages. In other parts of sub-Saharan Africa, studies show that SBC exposure has positive effects on ITN use in a variety of populations, including women of reproductive age [10], mothers of children under five [15], and all household members [14, 18]. Moreover, two studies conducted in Nigeria show that SBC exposure had positive effects on ITN use among household heads and their spouses [6] and all household members [5].

Our results suggest that SBC interventions can play an important role in improving ITN use among children under five. Given these findings and the low coverage of malaria SBC message exposure in the country, Nigeria should strive to scale up SBC interventions as the country seeks to achieve universal LLIN coverage. Meeting this intervention coverage target by 2020 requires improved ITN use among children. Although the country has made progress in improving malaria intervention coverage over the past decade, current coverage is behind 2015 targets, and large gaps remain to achieve the 2020 targets. Among women of reproductive age, only 36% recalled exposure to messages about malaria in the 6 months preceding the survey [4], even though SBC efforts to support key malaria prevention and management interventions have been ongoing [3, 7, 8]. Furthermore, of those women who recalled hearing any messages, fewer than half recalled messages related to ITNs. The most commonly recalled message was that sleeping inside a mosquito net is important (39%), but only 9% recalled messages about who should sleep inside a mosquito net [4].

The Federal Ministry of Health has an advocacy, communication, and social mobilization (ACSM) guide, which was updated in 2014 [33, 34], to help standardize ACSM programming, of which SBC messaging is an integral component, and to ensure that ACSM activities align with the NMSP. The NMSP notes that more evidence-based, culturally sensitive, and suitable SBC materials are needed, but it also states that the most effective SBC strategies (e.g., music, drama, sports, competitions) for improving the uptake of malaria interventions must still be determined [3]. In assessing possible SBC interventions, it will be important to consider the context in which they will be implemented. For example, mass ITN distributions are obvious opportunities on which to capitalize for promoting ITN use, but how is ITN messaging best conveyed if there are not mass ITN distributions? More evidence is needed to better understand and inform scaling up such interventions concurrently with prevention and treatment interventions in the country, especially in light of the substantial investments being made in SBC interventions and the country’s important push toward malaria elimination.

### Limitations

Due to the lack of variation in recall of ITN use messages, a valid exposure index could not be created to examine a dose–response relationship between SBC exposure and ITN use. In addition, the NMIS did not capture the intensity or source of specific messages. The study was limited to assessing the relationship between the outcome of interest and select individual and household-level background characteristics available in the dataset, which taken from a secondary data source. There was also a potential recall bias among survey respondents because the recall period for having heard messages related to ITN use was 6 months.

## Conclusions

This study suggests that caregiver exposure to ITN-related malaria messages improves the use of ITNs among children under five. These positive effects can be leveraged to advocate for malaria SBC interventions as an integral part of Nigeria’s strategy to scale up prevention and treatment intervention coverage to meet its 2020 targets. However, more evidence is needed on the impact of specific SBC interventions to determine which are the most effective and scalable in Nigeria.

## Abbreviations

ACSM: advocacy, communication, and social mobilization
CI: confidence interval
DHS: Demographic and Health Surveys
FMOH: Federal Ministry of Health
IRB: institutional review board
ITN: insecticide-treated net
LLIN: long-lasting insecticide-treated net
NMIS: Nigeria Malaria Indicator Survey
NMSP: National Malaria Strategic Plan
OR: odds ratio
SBC: social and behaviour change
USAID: United States Agency for International Development
WHO: World Health Organization

## References

[1] WHO (2017). World malaria report 2017.

[2] World Health Organization (2015). World malaria report 2015.

[3] Federal Republic of Nigeria (2014). National malaria strategic plan 2014–2020.

[4] National Malaria Elimination Programme (NMEP), National Population Commission (NPopC), National Bureau of Statistics (NBS), and ICF International. 2016. Nigeria Malaria Indicator Survey 2015. Abuja, Nigeria, and Rockville, Maryland, USA: NMEP, NPopC, and ICF International.

[5] Kilian A, Lawford H, Ujuju CN, Abeku TA, Nwokolo E, Okoh F (2016). The impact of behaviour change communication on the use of insecticide treated nets: a secondary analysis of ten post-campaign surveys from Nigeria. Malar J..

[6] Russell CL, Sallau A, Emukah E, Graves PM, Noland GS, Ngondi JM (2015). Determinants of bed net use in southeast Nigeria following mass distribution of LLINs: implications for social behavior change interventions. PLoS ONE.

[7] President’s Malaria Initiative. Nigeria malaria operational plan FY 2017. 2017. https://www.pmi.gov/docs/default-source/default-document-library/malaria-operational-plans/fy17/fy-2017-nigeria-malaria-operational-plan.pdf?sfvrsn=6. Accessed 11 Apr 2018.

[8] President’s Malaria Initiative. Nigeria malaria operational plan FY 2015. 2015. https://www.pmi.gov/docs/default-source/default-document-library/malaria-operational-plans/fy-15/fy-2015-nigeria-malaria-operational-plan.pdf?sfvrsn=6. Accessed 11 Apr 2018.

[9] Umeano-Enemuoh JC, Uzochukwu B, Ezumah N, Mangham-Jefferies L, Wiseman V, Onwujekwe O (2015). A qualitative study on health workers’ and community members’ perceived sources, role of information and communication on malaria treatment, prevention and control in southeast Nigeria. BMC Infect Dis.

[10] Boulay M, Lynch M, Koenker H (2014). Comparing two approaches for estimating the causal effect of behaviour-change communication messages promoting insecticide-treated bed nets: an analysis of the 2010 Zambia malaria indicator survey. Malar J..

[11] Macintyre K, Littrell M, Keating J, Hamainza B, Miller J, Eisele TP (2012). Determinants of hanging and use of ITNs in the context of near universal coverage in Zambia. Health Policy Plan..

[12] Mugisa M, Muzoora A (2012). Behavioral change communication strategy vital in malaria prevention interventions in rural communities: Nakasongola district, Uganda. Pan Afr Med J..

[13] Bowen HL (2013). Impact of a mass media campaign on bed net use in Cameroon. Malar J..

[14] Elder JP, Botwe AA, Selby RA, Franklin N, Shaw WD (2011). Community trial of insecticide-treated bed net use promotion in southern Ghana: the Net Use Intervention study. Transl Behav Med..

[15] Elmosaad YM, Elhadi M, Khan A, Malik EM, Mahmud I (2016). Communication for behavioural impact in enhancing utilization of insecticide-treated bed nets among mothers of under-five children in rural North Sudan: an experimental study. Malar J..

[16] Keating J, Hutchinson P, Miller JM, Bennett A, Larsen DA, Hamainza B (2012). A quasi-experimental evaluation of an interpersonal communication intervention to increase insecticide-treated net use among children in Zambia. Malar J..

[17] Polec AL, Petkovic J, Welch V, Ueffing E, Tanjong Ghogomu E, Pardo Pardo J (2015). Strategies to increase the ownership and use of insecticide-treated bednets to prevent malaria. Cochrane Database Syst Rev..

[18] Kilian A, Balayo C, Feldman M, Koenker H, Lokko K, Ashton RA (2015). The effect of single or repeated home visits on the hanging and use of insecticide-treated mosquito nets following a mass distribution campaign–a cluster randomized, controlled trial. PLoS ONE.

[19] Owusu Adjah ES, Panayiotou AG (2014). Impact of malaria related messages on insecticide-treated net (ITN) use for malaria prevention in Ghana. Malar J..

[20] Adebayo AM, Akinyemi OO, Cadmus EO (2015). Knowledge of malaria prevention among pregnant women and female caregivers of under-five children in rural southwest Nigeria. PeerJ..

[21] Birhanu Z, Abebe L, Sudhakar M, Dissanayake G, Yihdego Y, Alemayehu G (2015). Access to and use gaps of insecticide-treated nets among communities in Jimma Zone, southwestern Ethiopia: baseline results from malaria education interventions. BMC Public Health..

[22] Bedford KJA (2014). Local barriers and solutions to improve care-seeking for childhood pneumonia, diarrhoea and malaria in Kenya, Nigeria and Niger: a qualitative study. PLoS ONE.

[23] Millar KR, McCutcheon J, Coakley EH, Brieger W, Ibrahim MA, Mohammed Z (2014). Patterns and predictors of malaria care-seeking, diagnostic testing, and artemisinin-based combination therapy for children under five with fever in Northern Nigeria: a cross-sectional study. Malar J..

[24] Mitiku I, Assefa A (2017). Caregivers’ perception of malaria and treatment-seeking behaviour for under five children in Mandura District, West Ethiopia: a cross-sectional study. Malar J..

[25] Arroz JA (2017). Social and behavior change communication in the fight against malaria in Mozambique. Rev Saude Publica.

[26] Hwang J, Graves PM, Jima D, Reithinger R, Kachur SP (2010). Knowledge of malaria and its association with malaria-related behaviors–results from the Malaria Indicator Survey, Ethiopia, 2007. PLoS ONE.

[27] Baume CARR, Woldehanna S (2009). Factors associated with use and non-use of mosquito nets owned in Oromia and Amhara Regional States, Ethiopia. Malar J.

[28] Iwuafor AA, Egwuatu CC, Nnachi AU, Ita IO, Ogban GI, Akujobi CN (2016). Malaria Parasitaemia and the use of insecticide-treated nets (INTs) for malaria control amongst under-5 year old children in Calabar, Nigeria. BMC Infect Dis..

[29] Ovadje L, Nriagu J (2016). Multi-dimensional knowledge of malaria among Nigerian caregivers: implications for insecticide-treated net use by children. Malar J..

[30] StataCorp LLC. Logistic—logistic regression, reporting odds ratios. Stata base reference manual release 14. Stata press; 1985–2015. p. 1256–68.

[31] McCullagh P (1980). Regression models for ordinal data. J R Stat Soc Series B Stat Methodol..

[32] The DHS Program: Wealth Index. http://www.dhsprogram.com. Accessed 12 Mar 2019.

[33] Nigeria Federal Ministry of Health National Malaria Elimination Programme. Guidelines for malaria advocacy, communication and social mobilisation programmes; 2014.

[34] Nigeria Federal Ministry of Health National Malaria Control Programme. Advocacy, communication and social mobilisation strategic framework and implementation plan; 2010.

